# Role of the *Mosaic* Cisternal Maturation Machinery in Glycan Synthesis and Oncogenesis

**DOI:** 10.3389/fcell.2022.842448

**Published:** 2022-04-06

**Authors:** P. Sahu, A. Balakrishnan, R. Di Martino, A. Luini, D. Russo

**Affiliations:** Institute for the Experimental Endocrinology and Oncology, National Research Council of Italy, Naples, Italy

**Keywords:** cisternal maturation, onco-glycans, GOLPH3, Golgi apparatus, glycosylation

## Abstract

Tumorigenesis is associated with the deregulation of multiple processes, among which the glycosylation of lipids and proteins is one of the most extensively affected. However, in most cases, it remains unclear whether aberrant glycosylation is a cause, a link in the pathogenetic chain, or a mere consequence of tumorigenesis. In other cases, instead, studies have shown that aberrant glycans can promote oncogenesis. To comprehend how aberrant glycans are generated it is necessary to clarify the underlying mechanisms of glycan synthesis at the Golgi apparatus, which are still poorly understood. Important factors that determine the glycosylation potential of the Golgi apparatus are the levels and intra-Golgi localization of the glycosylation enzymes. These factors are regulated by the process of cisternal maturation which transports the cargoes through the Golgi apparatus while retaining the glycosylation enzymes in the organelle. This mechanism has till now been considered a single, house-keeping and constitutive function. Instead, we here propose that it is a mosaic of pathways, each controlling specific set of functionally related glycosylation enzymes. This changes the conception of cisternal maturation from a constitutive to a highly regulated function. In this new light, we discuss potential new groups oncogenes among the cisternal maturation machinery that can contribute to aberrant glycosylation observed in cancer cells. Further, we also discuss the prospects of novel anticancer treatments targeting the intra-Golgi trafficking process, particularly the cisternal maturation mechanism, to control/inhibit the production of pro-tumorigenic glycans.

## Introduction

Cancer is a multifaceted disease characterized by uncontrolled proliferation of aberrant cells that can develop in diverse organs and tissues and then invade other parts of the body (Chambers et al., 2002; Hanahan and Weinberg, 2011). Despite advances in cancer research over the last few decades, efficient treatment for several cancers in many cases is still lacking. One of the reasons for this is the multifactorial nature of the disease, determined by genetic, epigenetic, and transcriptional deregulation combined with environmental insults (Hanahan and Weinberg, 2011; Almendro et al., 2013). This heterogeneity not only exists among different cancer types but also within the same cancer type. As a result, current approaches are evolving towards precision medicine, aimed at finding novel markers for patient stratification. To date, the treatments approved for anticancer therapy are based either on relatively non-specific chemotherapy drugs or on the selective targeting of biological processes such as chromatin remodelling, DNA repair, proteasomal degradation, immune surveillance or aberrant kinases involved in signal transduction (Zwick et al., 2002; Lapenna and Giordano, 2009). However, a large fraction of such treatments suffers from drug resistance and tumor relapse, thus motivating the identification of new molecular drug targets. In this review, we focus on the prospect of novel anticancer treatments that impact on intra-Golgi traffic and, in particular, on the cisternal maturation machinery (CMM) that controls intra-Golgi transport, and hence glycosylation.

## Glycosylation in Cancer

Glycosylation is extensively altered during oncogenic transformation (Hakomori, 2002; Stanley, 2011; Pinho and Reis, 2015; Varki, 2017). Glycans are abundant polymers constituting the most abundant post-translational modifications of proteins and lipids, impacting a variety of biological functions (Varki and Gagneux, 2017). As compared to other processes, the role of glycosylation has been overlooked for several years in cancer biology and therapy. Analysis of glycans compared to other biopolymers is inherently complex due to their branched nature; moreover, this problem is compounded by the template-independent nature of their biosynthesis (Narimatsu et al., 2019; Schjoldager et al., 2020). As a result, glycan research often faces difficulties that can impede the development of projects by groups lacking full technical and scientific expertise in this area. Further, the contribution of aberrant glycans to tumorigenesis has not been clearly defined. Altered glycans have been used as tumor markers for several decades, e.g., carcinoembryonic antigen (CEA) 125, CEA19-9 (sLeA), and CEA72-4 (sTn) which are tumor-associated circulating serum O-glycans (Pinho and Reis, 2015). In most cases, it is unclear whether these aberrant glycans have a direct role in tumorigenesis, or they only act as intermediate players in the disease pathways initiated by other well-defined oncogenes. However, some studies have demonstrated how aberrant glycans can be “active players” in promoting oncogenesis (Nguyen et al., 2017; Rizzo et al., 2021). Truncated O-glycans frequently found in cancers have been observed to promote tumorigenesis by inducing EGFR and ErbB2 receptor activation (Freitas et al., 2019) and activation of MMP14 that increases the degradation of extracellular matrix (ECM) and initiates tumor growth in mice liver (Nguyen et al., 2017). Along the same line, glycan changes occurring in the later stages of cancer that were considered as “passive players” are emerging as determinants of drug resistance. For instance, increase in surface sialylation of N-glycans linked to plasma membrane (PM) receptors were shown to induce resistance to doxorubicin by acting as a barrier to chemotherapeutic agents (Ma et al., 2013; Ji et al., 2017). These glycan aberrations participate in tumorigenesis, and it is, therefore, conceivable that they may result from potential oncogenes acting upstream to the observed malignant phenotypes. Currently, there is no clear experimental evidence for glycosylation-based oncogenic mechanisms. As a basis for identifying errors in glycan biosynthesis and understanding how they occur, it is necessary to comprehend the underlying mechanisms of glycosylation.

## Glycosylation Within the Golgi Apparatus

**FIGURE 1:**
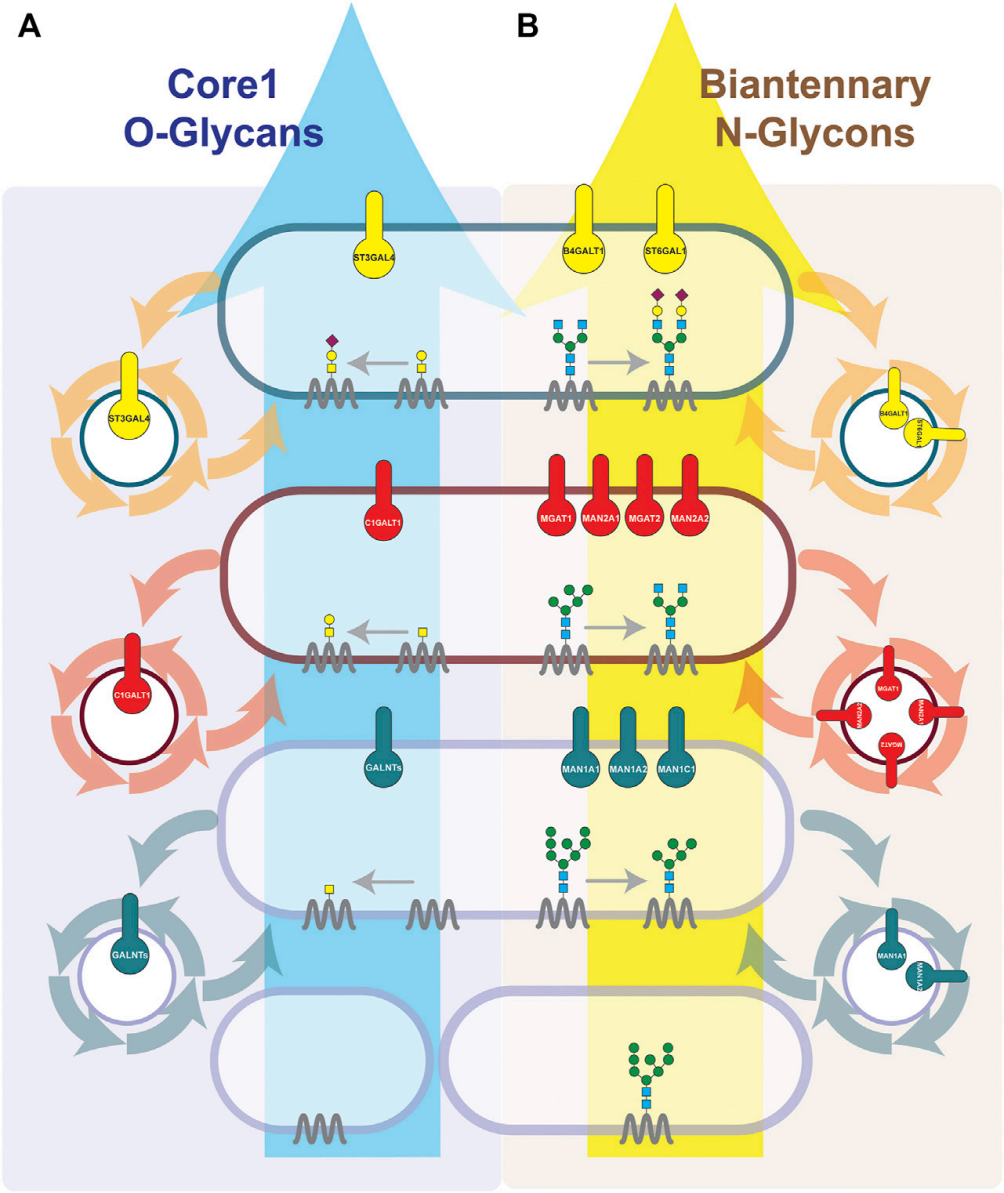
Cisternal maturation controls the positioning of different subsets of glycoenzymes within each Golgi cisterna. The synthesis of Core-1 O-glycans **(A)** and Biantennary N-glycans **(B)** is depicted. We represent how the cisternal maturation machineries (COPI-dependent or COPI-independent) by controlling the recycling of each serial acting enzymes regulate their localization in the *cis*-, medial- or *trans*-Golgi and define the final glycosylation outcome. Sequentially acting enzymes are known to interact with each other and form an enzymatic module within the same cisterna (Khoder-Agha et al., 2019). Enzymes are known to be enriched in 1–2 cisterna, with some enzymes having a sharper localization then others (Tie et al., 2018; Pothukuchi et al., 2021). We have represented them in single cisterna for communication purposes. Briefly, O-glycosylation occurs solely at Golgi. O-linked glycosylation starts at *cis-*Golgi by adding GalNAc to Ser/Thr residues of a cargo protein. Then, in the medial, based on the availability of glycosyltransferases, galactose or GlcNAc are added, and in the case of core-1 O-glycan, the galactose is added. Finally, sialic acid is added by sialyltransferase present at the *trans-*Golgi. Unlike O-glycosylation, N-glycan biosynthesis starts at ER. Indeed, the cargos which arrive at the Golgi have already undergone the addition of a block of 14 sugars, from which four glycans have been trimmed. In the *cis-*Golgi, further three mannoses are removed by alpha-mannosidases as MAN1A1, MAN1A2 and MAN1C1. Then in medial-Golgi, upon the trimming of two more mannose residues by MAN2s, GlcNAc is added by MGAT1 and MGAT2, and finally when cargo reaches the *trans-*Golgi, galactosylation and final sialylation cap are added.

Cargoes are synthesized in the endoplasmic reticulum (ER) and then transported to the Golgi apparatus. Except for the cargoes that undergo N-glycosylation which experience an initial step of glycan addition before leaving the ER, most of the glycosylation pathways occur in the Golgi apparatus, (Varki and Gagneux, 2017). The Golgi is endowed with approximately 200 resident glycosyltransferases (glycoenzymes) (Varki, 2017), which catalyse the serial addition of sugars to the cargo in the lumen of the Golgi apparatus (Stanley, 2011). These enzymes are arranged in orderly succession along the Golgi cisternae, mirroring the order in which they act in elongating the growing glycan chains. Moreover, each Golgi cisterna is equipped to carry out a complex set of glycosylation reactions and thus to act as a specialized reaction chamber. Enzymes are enriched in 1–2 cisternae, and it is clear that some enzymes have a sharper localization than others (Tie et al., 2018; Pothukuchi et al., 2021). Within each cisterna, subsets of glycoenzymes are organized to catalyse serial reactions of the same glycosylation pathway, generating a functional processing module (Narimatsu et al., 2019) (Figure 1). The processing occurs during transport through Golgi cisterna, where cargo encounters glycoenzymes often organized in metabolic modules, and single monosaccharide units are sequentially added, which defines the cargo-specific sugar configuration or glycoform (Schjoldager et al., 2020) (Figure 1). Specific glycoenzymes compete for the same substrates, which may contribute to the formation of different glycosylation pathways and of glycoforms (variants of glycosylation chains). Glycosylated cargoes then exit from the *trans-*Golgi where they are sorted to the appropriate cellular destination. Hundreds of different glycoforms have been found linked to cargoes (Moremen and Haltiwanger, 2019). The glycoform repertoire of a given cell type is not a random collection of all possible synthesizable configurations, instead, they fall into a discrete distribution of variants, suggesting that the glycosylation process is highly regulated. Several factors have been proposed to regulate the overall glycan synthesis process, and the interested reader can refer to Pothukuchi et al. (2019). As the transcript level of different glycoenzymes does not quantitatively correlate with the glycans synthesized by a cell (Moremen and Haltiwanger, 2019), the intra-Golgi localization, and the control of enzyme levels by their degradation rate in lysosome or plasma membrane, have emerged as a new theme for regulating glycan synthesis (Pothukuchi et al., 2019). Both the levels and positioning of the enzymes is controlled by membrane transport mechanisms operating in the Golgi. Since the glycoenzymes are “single pass” membrane proteins with a short cytosolic tail and the catalytic domain-oriented towards the Golgi lumen, their levels and intra-Golgi positioning is indirectly controlled by proteins peripherally associated with the cytosolic side of the Golgi. Such peripherally associated Golgi proteins control the dynamics of glycoenzymes and constitute the components of the CMM (Tu et al., 2008; Rizzo et al., 2021). The process of cisternal maturation is broadly accepted as the predominant mechanism of intra-Golgi trafficking and is supported by solid experimental evidence in various cell types including mammalian and yeast cells (Bonfanti et al., 1998; Bonifacino and Glick, 2004; Rizzo et al., 2013). Transport by cisternal maturation represents the fundamental framework within which the glycosylation of most proteins and lipids are regulated at the Golgi apparatus.

## Cisternal Maturation is a *Mosaic* of Recycling Pathways

**FIGURE 2:**
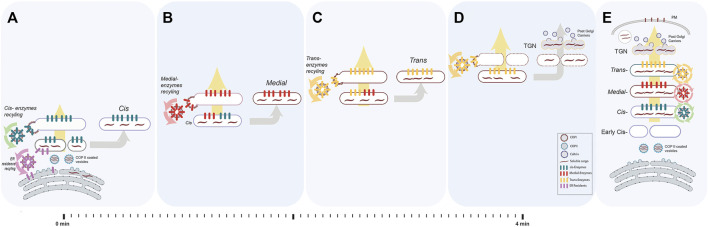
The process of cisternal progression and maturation. We depicted an ideal and a simplified version of the cisternal maturation model for communication purposes. In this model, each enzyme recycle from the cisterna in which resides to the proximal cisterna within COPI-vesicles. Evidence from yeast shows that there are alternative recycling steps that use COPI-independent vesicles. Briefly, the Golgi in mammals usually comprises 3–6 stacked cisterna (*cis*, medial and *trans*). **(A)** Once the cargo (dark maroon waves) arrives from ER in COPII coated vesicles (cyan coated round profiles containing short dark maroon waves representing cargoes), they fuse and form early *cis-*compartment. At the same time, *cis*-resident glycoenzymes (green bars) are recycled back (green arrows) from pre-existing *cis* to the newly forming *cis* in COPI coated vesicles contributing to formation of the new *cis*-cisterna. There, ER resident proteins (pink bars) and membranes are recycled back to the ER *via* COPI-dependent vesicles (pink arrows). At the same time, a *trans*-cisterna disassembles into anterograde carriers **(D)**. **(B)** The newly formed *cis-*cisterna receives the medial-enzymes (red bars) which are recycling (red arrows) from the pre-existing medial-cisterna to form a *cis/medial* hybrid. From this hybrid compartment, the *cis-*glycoenzymes (green bars) are recycled back (green arrows) to a new arriving *cis-*compartment (as in **A**) and the medial-glycoenzymes (red bars) are the only one to be left in the mature medial-cisterna. **(C)** Similarly, the newly formed medial-cisterna receives the *trans*-glycoenzymes (yellow bars) which are recycling (yellow arrows) from the pre-existing *trans*-cisterna to form a medial*/trans* hybrid. From this hybrid compartment, the medial-glycoenzymes (red bars) are recycled back (red arrows) to a new formed medial-compartment (as in **B**) and the *trans*-glycoenzymes (yellow bars) are the only one to be left in the mature *trans*-cisterna. **(D)** Through all the steps, the cargo never leaves the cisterna rather, it progresses anterogradely (large yellow arrow) along with maturing cisterna. Glycoenzymes (represented as coloured bars specific for each cisterna) also move forward within progressing cisterna, but, once reached the appropriate intra-Golgi compartment, are retrieved, and recycled *via* COPI coated vesicles, driving the inter-conversion of the *cis*-cisterna into medial*-* and *trans*-elements (as in **A–C**). Once cargo reaches the *trans*-face of Golgi, the TGN will be disintegrated into the post-Golgi clathrin coated vesicles containing cargo. **(E)** This happens in synchrony with cisternal progression. As a result, the cargoes moving forward are successively exposed to the enzymatic complement of each cisterna. The balance between the opposing anterograde and retrograde membrane fluxes results in the cycling of enzymes between two specific cisterna (yellow circle indicates recycling from the *trans-* to the medial-cisterna, red circle from the medial- to the *cis*-cisterna, green circle from the *cis* to the new forming *cis*-cisterna and pink arrows from the newly formed *cis*-cisterna to the ER) and determines the sub-Golgi localisation of the glycoenzymes.

**FIGURE 3:**
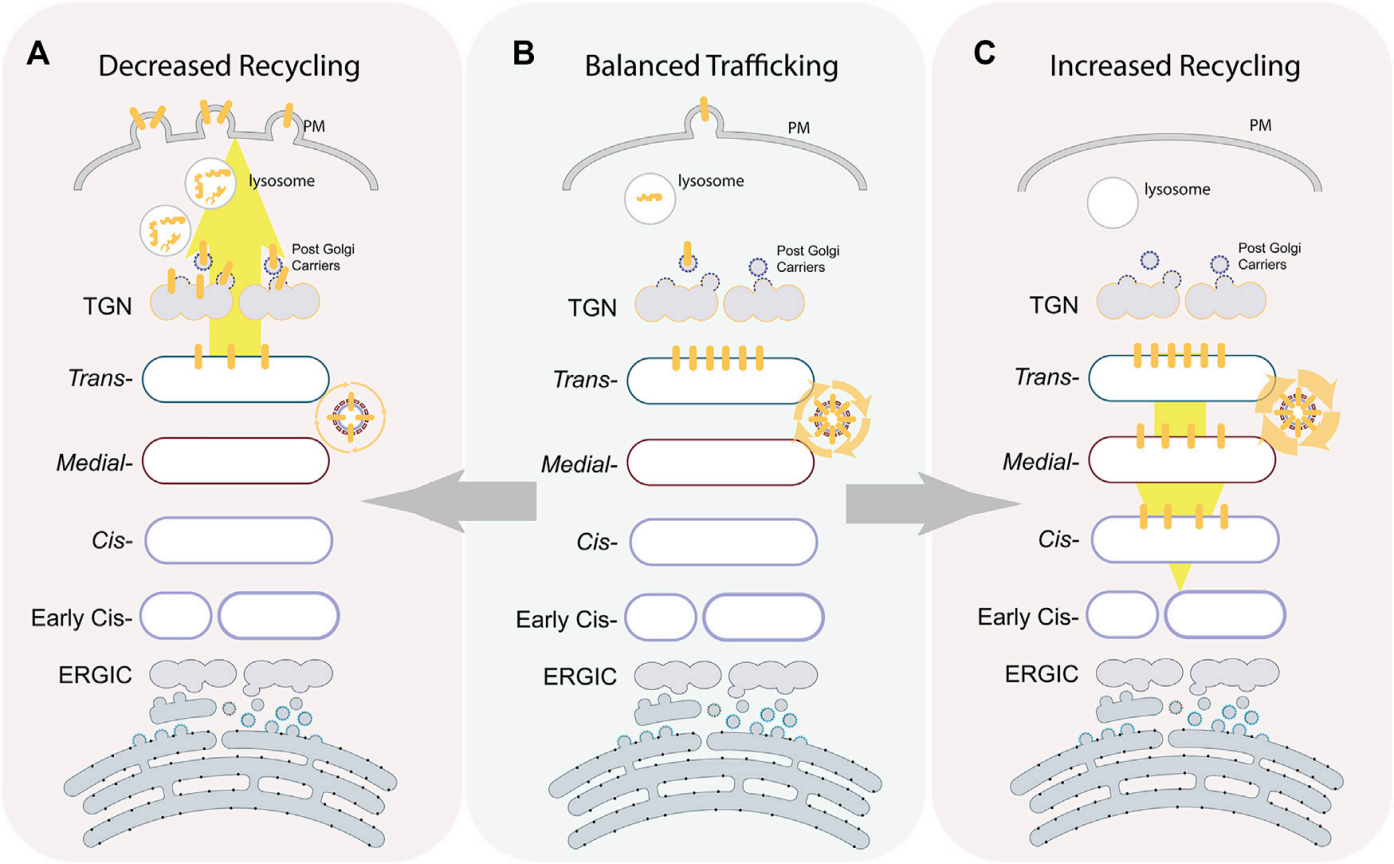
Intra-Golgi recycling controlling glycoenzymes positioning and levels. The illustration depicts the localization of a prototypic *trans-*glycoenzymes (yellow bars) at different states. At a steady state maturation, machinery maintains the enzymes to localize to a specific sub-Golgi compartment by continuous recycling **(B)**. Overexpression of components of the recycling machinery increase in intra-Golgi retrograde transport of the enzymes that redistribute the enzymes back in the whole Golgi **(C)**. On the other hand, depletion of components of the recycling machinery results in a loss of enzymes from the Golgi toward lysosomes or PM where they undergo degradation or ectodomain shedding, respectively **(A)**.

The Golgi complex is composed of flattened, stacked membranous cisterna with *cis-* to *trans-*polarity (Varki, 2017) (Figure 2). Each Golgi cisterna is defined by its Golgi residents. The cisternal maturation mechanism proposes that new cisternae continuously form on the *cis-* face of the Golgi (by the homotypic fusion of vesicles coming from the ER) and disassemble at the *trans*-face, generating a flow of membranes from the *cis-* to the *trans-*Golgi up to the PM. Cargo proteins remain in the progressing cisterna until they leave the Golgi in post-Golgi carriers for their final destinations. At the same time, the cisternae change their “identity” (see below) from *cis* to medial to *trans*. Otherwise, glycoenzymes also move anterogradely along with the progressing cisterna for a given distance, but they do not leave the Golgi. When they reach the (*cis*, medial or *trans*) where they must reside, they are retained there by a mechanism based on active recycling via sorting in retrograde transporting vesicles. Thus, instead of moving forward like the cargoes, glycoenzymes are transported in vesicles to acceptor proximal compartments, to balance the anterograde flow of progression. Once incorporated into the acceptor cisterna, retainer adaptors such as GRASP55 prevent a further round of glycoenzyme recycling. The retaining action is required for dynamic compartmentalization of specific glycoenzymes within the Golgi compartment (Pothukuchi et al., 2021). The balance between anterograde movement of the cisterna and the retrograde recycling, along with the retention activity of retainers help to determine the localization of the enzymes in the stack (see below). The CMM, besides determining the positions of glycoenzymes in the Golgi apparatus also contributes substantially to controlling the level of glycoenzymes. At steady-state, a fraction of glycoenzymes constantly escapes the recycling mechanisms and is transported for lysosomal degradation, or to the PM where they are degraded by ectodomain shedding (Sun et al., 2021). This fraction is replenished by new synthesis, thus ensuring constant levels of each enzyme. However, if the recycling mechanisms of the glycoenzymes lose efficiency or slow down, the fraction of enzymes exported to the lysosomes or PM and degraded increases, thus decreasing the overall enzyme level in Golgi. Conversely, if the recycling mechanism is potentiated, the fraction of enzymes degraded over time decreases or is completely inhibited. As a consequence, enzyme levels increase, even massively, altering the molecular and functional profile of the glycans (Figure 3) (Rizzo et al., 2021). Thus, the intra-Golgi transport mediated by cisternal maturation controls both the levels and position of glycosylation enzymes in the Golgi.

The retrograde recycling of the enzymes required for cisternal maturation is mediated mainly by the Coat protein I (COPI) complex. Indeed, mutation of a COPI subunit can impair the maturation of cisterna as observed in yeast cells (Matsuura-Tokita et al., 2006). Recent studies have described multiple COPI dependent and independent pathways operating at the Golgi (see below) to promote enzyme recycling (Tu et al., 2008; Liu et al., 2018; Witkos et al., 2019; Zhao et al., 2019; Casler et al., 2021; Rizzo et al., 2021; Sardana et al., 2021), leading to a recent proposal that cisternal maturation is driven not by a single pathway but a mosaic of several likely independent recycling pathways (Rizzo et al., 2021).

Initial models of cisternal maturation had hypothesized a COPI dependent recycling of the enzymes within the Golgi likely mediated by their direct interaction with the COPI complex (Glick et al., 1997). Indeed, a differential affinity of enzymes to the COPI coat was hypothesized to regulate the differential localization of enzymes along the cisterna. Further direct binding of at least some enzymes to the COPI coat has been described (Liu et al., 2018). Later studies in yeast proposed a variation to this general theme by identifying for the first time an adaptor, Vps74, that links the enzymes to the COPI coat. Vps74 binds to a specific motif present in the cytosolic tails of glycosylation enzymes and simultaneously binds to the COPI coat through its N-terminus (Tu et al., 2012). Thus, by bridging the coat with the client glycoenzymes, this recycling adaptor allows the recruitment of a specific subset of glycosylation enzymes into the COPI recycling pathway. Golgi Phosphoprotein 3 (GOLPH3), the human Vps74 homolog, was shown to act on a group of glycosylation enzymes among which there is a subset of enzymes which catalyse serial reactions responsible for the synthesis of specific glycosphingolipids (GSLs) and which represent an enzymatic module (Rizzo et al., 2021). The evidence supports a model by which the localization of GOLPH3 clients in the *trans-*Golgi cisterna depends on the localization of GOLPH3 itself in the same cisterna. GOLPH3 localization is, in turn, determined by the presence in the *trans*-cisterna of the phosphatidylinositol 4-phosphate [PI(4)P] (Wood et al., 2009; Rahajeng et al., 2019). PI(4)P is a *trans-*Golgi identity marker produced by the PI4KIIIβ kinase, by which GOLPH3 is recruited to the membrane. Thus, at each transport round, GOLPH3 is recruited to the *trans*-Golgi by PI(4)P (Dippold et al., 2009). Once recruited, GOLPH3 incorporates its client enzymes into COPI-dependent retrograde vesicles for transport to the proximal cisterna (presumably the maturing medial-elements) (Eckert et al., 2014). In this cisterna*,* PI(4)P is degraded, causing GOLPH3 to detach and release the GOLPH3 clients in the (medial) cisternal membrane. At the same time the *trans*-cisterna disassembles and the medial-cisterna completes maturation into a *trans*-element, where PI(4)P is produced again, and GOLPH3 is recruited to start a new transport round. In this scheme, glycoenzyme localization is driven by the production of identity markers [e.g., PI(4)P] on maturing cisterna, which is itself driven by the Rab conversion process (see below); and the GOLPH3 clients cycle between *trans-* and medial-cisterna, with a prevalent steady-state localization in the *trans*-Golgi because of the underlying synthesis and degradation cycle of PI(4)P in the *trans-* and medial-cisterna, respectively. Similar mechanisms likely function at other levels of the Golgi complex, and other components may be involved in the recruitment of recycling adaptors or directly COPI, but this remains to be tested experimentally. Indeed, yeast Erd1 was recently found to act as a transmembrane adaptor molecule to links client enzymes to COPI coat surprising by binding to the adaptor Vps74 (Sardana et al., 2021). Thus, the COPI-dependent recycling is a mosaic of at least three different pathways (one adaptor independent and two adaptor dependent pathways). There are likely to be other COPI-dependent pathways as for instance GORAB and Scyl1 were found to scaffold COPI coat on *trans*-Golgi to promote recycling of ST6GAL1 enzyme (Witkos et al., 2019). Whether they also act as a recycling adaptor is still to be understood. Of note, while we classify them as three different COPI pathways—it includes the possibilities of three distinct COPI vesicles, three distinct pathways of recruiting Golgi residents into the same COPI vesicle and all the variations in between. In addition to biochemical evidence, morphological evidence provided by Cryo-electron microscopy studies show how COPI-coated vesicles changed their luminal density, membrane thickness and size during progression through the Golgi stack, in a manner that reflects corresponding changes in the morphology of the donor cisterna (Bykov et al., 2017), supporting the idea of the existence of distinct intra-Golgi COPI vesicles serving particular transport routes.

In addition to COPI-dependent pathways, yeast studies have also shown the existence of COPI-independent recycling pathways especially operating at the *trans*-Golgi. For instance, a COPI-independent pathway mediated by Dop1 for recycling enzymes from *trans*-Golgi network (TGN) to Golgi was identified in yeast (Zhao et al., 2019). Furthermore, recycling of classes of enzymes in the late Golgi was shown to be mediated by the adaptor protein complex-1 (AP-1) clathrin adaptor along with Ent5, a clathrin adaptor. The AP-1/Ent5- mediates two sequential intra-Golgi recycling pathways that define the polarized distribution within Golgi of two classes of Golgi residents (Casler et al., 2021). Thus, there are at least three different COPI-independent recycling pathways shown to operate in the Golgi. Of note, multi-transmembrane proteins TM9SF2 and LAPTM4A were shown to be necessary for the Golgi localization of a subset of glycosylation enzymes (Yamaji et al., 2019). Whether these proteins act in a COPI-dependent or independent manner is not clear, even though TM9SF2 is equipped with a motif for interacting with COPI (Woo et al., 2015; Yamaji et al., 2019). In general, it is becoming apparent that there is still a lot of intra-Golgi recycling pathways to discover.

While these multiple recycling pathways at the Golgi still act within the cisternal maturation framework of intra-Golgi transport, they change it in a subtle but important manner- the recycling of glycosylation enzymes is not mediated by single homogenous house-keeping pathway, but rather by a sophisticated mosaic of pathways that act in a coordinated manner and can likely be subjected to independent regulatory controls to precisely build the glycan profile of the cell.

## The Molecular Machinery of Cisternal Maturation Reflects its Mosaic Nature

The mammalian Golgi stack is thought to consist of three distinct compartments - *cis*-, medial- and *trans*-Golgi and so the transport of cargoes from *cis* to *trans*-Golgi will require at least three distinct maturation steps, each with its set of above-mentioned molecular components (Figures 1, 2). Of note in yeast recent studies propose there could just be three steps in this maturation scheme that are required to transport cargoes from *cis* to *trans*-Golgi (Day et al., 2013). But when we look at the number of molecular components available in the Golgi, it does not necessarily reflect the three-step pathway. For instance, while one could expect the requirement of three distinct Rab proteins corresponding to the three distinct maturation steps, we find close to two dozen Rab proteins localized to the Golgi apparatus (Barr, 2009; Thomas and Fromme, 2020), which is an order of magnitude more than the minimum required. Similarly, there are more than expected number of Golgin tethers. Biochemical evidence shows how *cis*-localised GM130 tethers vesicles originating from ER, while TMF, Golgin-84 and GMAP210 mediate different intra-Golgi transport routes. GMAP210 and golgin-84 tether preferentially a population of presumably COPI vesicles originating from medial-Golgi which could dock with the early Golgi compartments, while TMF tethers late derived COPI vesicles, which dock with the medial-Golgi compartment. A similar situation is present with other tethers in other districts in the Golgi (Wong and Munro, 2014). It is to be noted this excess number of Rabs and tethers is still valid if we remove the isoforms of the same gene from consideration. Similarly, even the COPI coat subunits have distinct isoforms and that are distributed non-uniformly over the Golgi stack (Moelleken et al., 2007). Whether this corresponds to distinct specificities for the Golgi residents incorporated into the vesicles is yet to be resolved. While at first sight it might seem confounding, we propose that this goes in line with the proposal that the cisternal maturation is a coordinated event of multiple maturation pathways each characterized by a distinct Rab, glycoenzyme adaptors and possibly tethering molecules. The multiple pathways may operate completely distinctly or converge into the same COPI vesicle. In either case, the existence of this mosaic of maturation pathways suggests a potential for specific regulation of a subset of glycosylation enzymes.

This hypothesis has an important implication in oncology, since it changes the perspective of cisternal maturation from house-keeping pathway that can barely be altered without dire consequences to the cell to a complex mosaic of pathways each when altered affecting only a specific set of enzymes but not the cisternal maturation and cargo transport as such. This can potentially lead to specific alterations in glycoenzyme levels and their intra-Golgi positioning with a subsequent change in the glycan profile of the cell. We discuss the potential of the CMM components to contribute to oncogenesis from this mosaic maturation point of view.

## Candidate Glyco-Oncogenes of the Cisternal Maturation Machinery

Several studies have underlined how alterations of glycoenzyme trafficking affect their position inside the Golgi cisterna, affecting the sequence of glycosylation reactions and the final cargo-specific glycan outcome (Chia et al., 2019; Rizzo et al., 2021). In this scenario, we have recently focused on identifying plausible oncogenes operating within the mechanism of glycoenzyme trafficking. We have particularly focused on the Golgi protein GOLPH3, a well-known oncogene (Scott et al., 2009). When amplified in certain tumors, GOLPH3 exerts its oncogenic activity by influencing glycan biosynthesis (see below). GOLPH3 is thus the first demonstrated glyco-oncogene. Similarly, several CMM genes that could support and drive tumorigenesis through altered glycan synthesis are over-expressed due to gene amplification in several solid tumors (Figure 5). Thus, the alteration of expression in CMM genes could support oncogenesis by altering the dynamics and levels of relevant glycoenzymes. These findings imply that previously unknown oncogenes may be hidden in the CMM or glycoenzyme genes. These genes have yet to be discovered or simply overlooked, despite being part of the glycan synthesis machinery, even though they are not glycoenzymes. Indeed, current therapies aim at cancer glycans exposed on cancer cells, rather than targeting potential oncogenes involved in their underlying synthetic machinery. To date, only a limited number of glycoenzymes inhibitors are currently being evaluated as anticancer drugs in clinical trials, such as 2-Fluorofucose (2FF), which is a fucosylation inhibitor used alone or in combination with pembrolizumab (anti-PD1) to treat specific carcinomas (NCT02952989). Our goal is to develop a framework for the analysis of the oncogenic potential of CMM genes and reveal novel Golgi-related oncogenes. Several preclinical studies highlight the active role of these genes in tumorigenesis through the regulation of trafficking and other cellular functions. Below, we discuss the oncogenic potential of different categories of CMM genes, found frequently amplified in cancers. We do not attempt to exhaustively survey the potential glyco-oncogenes. Rather we wish to discuss the possible oncogenic mechanism of action, due to intra-Golgi glycoenzyme recycling and synthesis of pro-tumorigenic glycoforms, of a limited number of prototypes belonging to the main classes of Golgi machinery proteins.

## Glycoenzyme Adapters: Central Players in Glycan Biosynthesis

Two main types of adapters have been described so far, the recyclers and the retainers. In the following we will define their mechanism of action based on the knowledge acquired about the best characterized of these types of proteins.

### The Prototype of Recycling Adapters: GOLPH3

**FIGURE 4:**
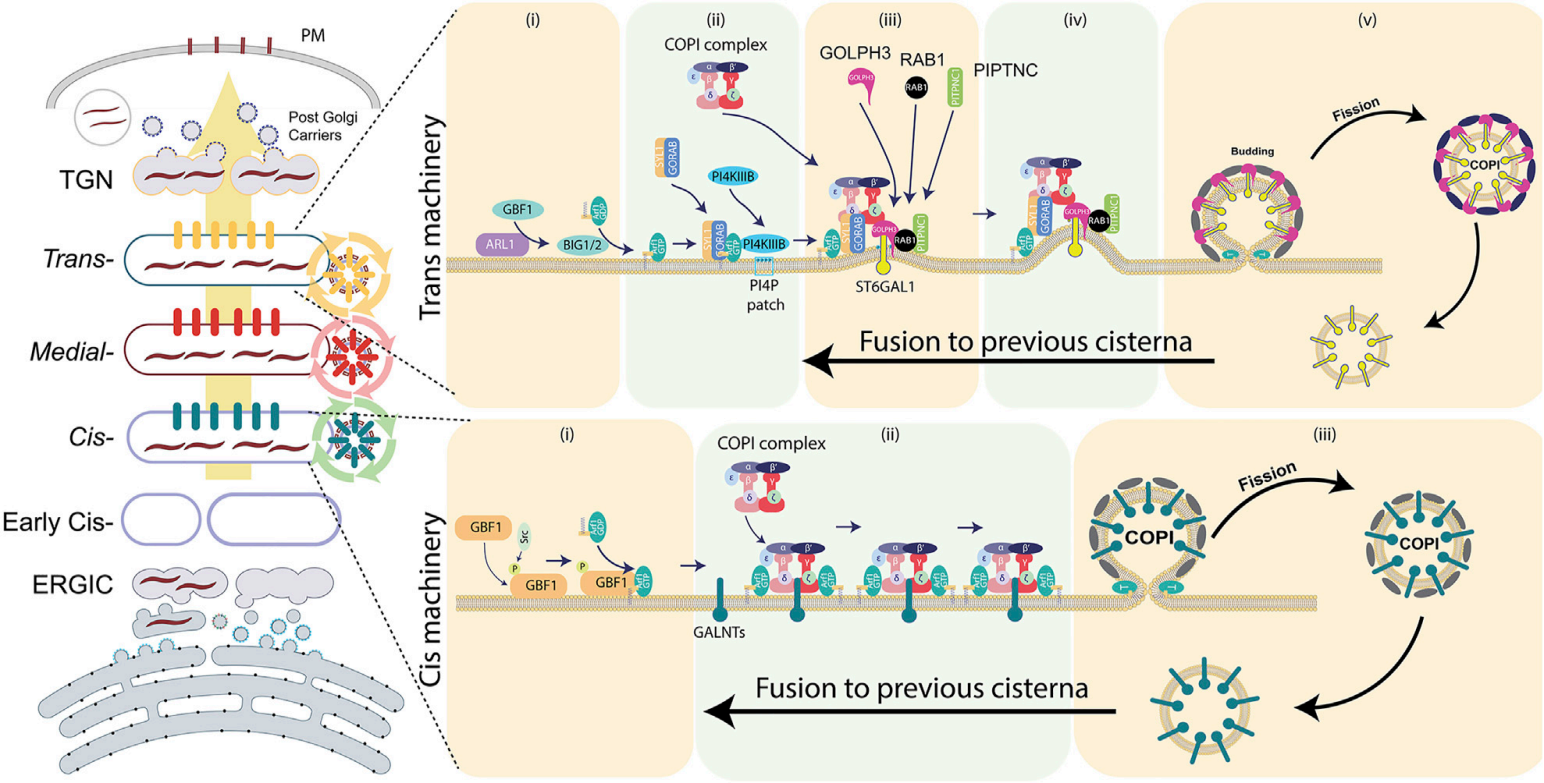
Components of the COPI-dependent recycling machinery at the *cis-* and *trans-*Golgi faces. Several of components of CMM found to be amplified in solid tumors. For further details, refer to the main text.

GOLPH3 is a protein located in the late Golgi and TGN with a variety of functions (Buschman et al., 2015; Rizzo et al., 2017). GOLPH3 is required for Golgi ribbon structure maintenance, vesicle trafficking and Golgi glycosylation (Ng et al., 2013; Rizzo et al., 2021). GOLPH3 facilitates vesicular trafficking from TGN to PM, with the aid of MYO18A and PITPNC1 (Halberg et al., 2016; Rahajeng et al., 2019). In response to DNA damage, GOLPH3 is phosphorylated by DNA damage protein kinase (DNA-PK), which induces Golgi dispersal and bypasses cell apoptosis (Farber-Katz et al., 2014). Moreover, several studies shown that GOLPH3 acts as an adaptor to promote recycling of a specific set of glycosylation enzymes by acting as a bridge between the enzymes and COPI and thus favouring the incorporation of glycoenzymes in COPI recycling vesicles (Tu et al., 2008). GOLPH3 gets recruited to Golgi by binding to PI(4)P through its PH domain (Wood et al., 2009). While the PI(4)P-dependent recruitment of GOLPH3 to the Golgi is common across yeast and mammalian systems, in *Drosophila melanogaster* Rab1b has been shown to be involved in GOLPH3 recruitment (Sechi et al., 2017), and similarly, Erd1 participates in the recruitment of Vps74, the GOLPH3 homolog in yeast (Sardana et al., 2021). Once recruited to membranes GOLPH3 binds to enzymes that bear the Lxx(R/K) motif in their cytosolic tails and promotes their entry into recycling COPI vesicles and thus retaining the enzymes in the *trans*-Golgi (Figure 4) (Tu et al., 2008; Rizzo et al., 2021). We have recently demonstrated that among the GOLPH3 clients there are several enzymes responsible for glycosphingolipids (GSL) biosynthesis such as lactosylceramide synthase (LCS, or B4GalT5), Gb3 synthase (Gb3S or A4GALT1), GM3 synthase (GM3S, or ST3GAL5), and GD3 synthase (GD3S, or ST8SIA1). Additionally, other enzymes belonging to proteoglycans (PGs) and N-glycans pathways also bear the GOLPH3 recognition motif, representing potential clients (Rizzo et al., 2021). Interestingly, a group of glycoenzymes located in *cis*-Golgi, and without the Lxx(R/K) binding motif, have also been reported to interact with GOLPH3. Moreover, one of these enzymes, GALNT2, recycles efficiently in a GOLPH3-dependent manner *in vivo* (Welch et al., 2021). How these enzymes are recognized and how GOLPH3 acts on *cis-*Golgi localized enzymes when it is itself localised at the *trans-*Golgi remains to be addressed. In addition to promoting sorting of specific enzymes into COPI vesicles, GOLPH3 by inserting a β-hairpin within the lipid bilayer induces a positive curvature of the Golgi membrane favouring the vesicles formation and scission (Rahajeng et al., 2019). Noteworthy, GOLPH3 undergoes extensive post-translational modification such as phosphorylation and ubiquitination, making its activity physiologically tuneable (Dippold et al., 2009; Farber-Katz et al., 2014; Zhou et al., 2014).

**FIGURE 5:**
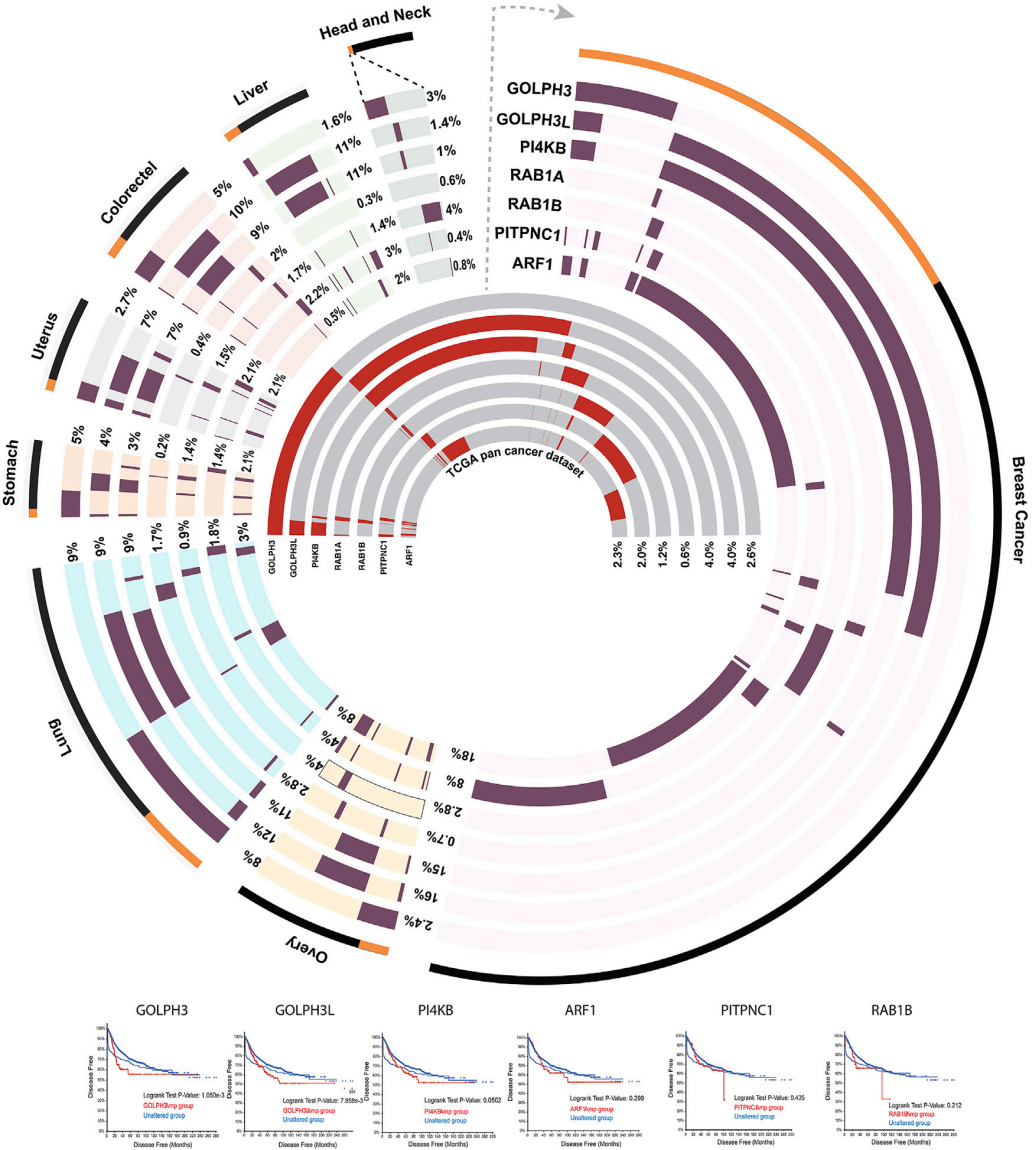
Circos plot (Krzywinski et al., 2009) showing the amplification frequency of seven CMM genes in the top eight cancer types in which they are more frequently amplified. The outermost layer showing the percentage of samples (orange) that have amplification in at least one of the seven CMM genes considered, compared to total number (black) of samples in the study. Next seven tracks showing the amplification frequency (dark maroon) of each gene in the individual cancer type considered. The seven inner most tracks represent amplification frequency (red) of each of the CMM gene considered across whole repertoire of 32 different pan cancers reported in The Cancer Genome Atlas (Gao et al., 2013).

GOLPH3 is a validated oncogene amplified across various cancer types (Figure 5) (Gao et al., 2013). It has been reported to be amplified in 8% of lung adenocarcinomas (Scott et al., 2009), 6% of ovarian cancer and 6% of bladder cancers (Cerami et al., 2012). GOLPH3 overexpression in tumors has been significantly correlated with worst prognosis and in the development of drug resistance (Ma et al., 2014; Wang et al., 2019; Yu et al., 2020). Indeed, in the recent years, GOLPH3 has evolved as a drug target in cancers which develop resistance to conventional anti-cancer therapies (Ye et al., 2019). GOLPH3 was found to act as an oncogene by promoting the Akt signalling in cancer cells (Scott et al., 2009). We recently showed that GOLPH3 promotes Akt signalling by altering specific GSL levels in cells through its action of the GSL biosynthetic enzymes in the Golgi (Rizzo et al., 2021). GOLPH3 silencing led to a loss of its client enzymes from the *trans*-Golgi to lysosomes where they are then degraded. Conversely, GOLPH3 overexpression, as observed in tumors, increases the levels of GOLPH3 clients by enhancing their intra-Golgi recycling. In addition, GOLPH3 induced increased recycling of the enzymes also promotes their relocalization toward *cis*-Golgi providing a positional advantage to the clients involved in competing reactions. The glycoenzymes involved in the GSL synthesis represent an excellent instance to explain this phenomenon. The glucosylceramide (GlcCer) is the common precursors of all GSLs and it is produced in the *cis*-Golgi. GlcCer then is galactosylated to produce lactosylceramide (LacCer), by LCS. Once produced, four enzymes compete for the further glycosylation of LacCer, which is the metabolic branch point for the formation of different classes of GSLs: B4GalnT1 produces GA2 the precursor of asialo series, GM3S produces GM3 the precursor of ganglio series, Gb3S produces Gb3 the precursor of globo series; and B3GnT5 produces Lc3 the precursor of lacto series. Since LCS, Gb3S and GM3S are GOLPH3 clients, but not B4GalnT1 and B3GnT5, the GOLPH3 overexpression relocates these enzymes toward *cis*-Golgi favouring the metabolic channelling of GlcCer towards the synthesis of ganglio and globo series GSL, rather than asialo and lacto series GSL. This competitive advantage coupled to increased levels of client enzymes in GOLPH3 expressing cells results in increased biosynthesis of specific GSLs. The GOLPH3-dependent oncogenic effect is then exerted by specific GSL species which most likely act on adhesion molecules, and Receptor Tyrosine Kinases (RTKs) promoting cancer growth and metastasis (Kovbasnjuk et al., 2005; Russo et al., 2016; Jacob et al., 2018; Russo et al., 2018; Furukawa et al., 2019; Rizzo et al., 2021). Similarly, another GOLPH3 client enzyme, ST6GAL1 has also been shown to contribute to its oncogenic effect by sialylating integrins (Seales et al., 2005). Thus, GOLPH3 acts as a rheostat to control the synthesis of a glycan repertoire that promotes cell growth and division by regulating the dynamics of its client enzymes (Rizzo et al., 2021).

The widespread amplification of GOLPH3 in solid tumors and associated worsened clinical prognosis makes it an ideal candidate for targeted therapy in solid tumors. The *in vivo* silencing of GOLPH3, in a combinatorial anti-tumor therapy, by using GOLPH3 siRNA and Gefitinib both conjugated to nanoparticles, inhibited EGFR signalling and glioma growth in the mouse model (Ye et al., 2019). Interestingly, the human GOLPH3 paralog, GOLPH3-like (GOLPH3L), which has a 79% similarity with GOLPH3, offers an additional therapeutic target since it shares its repertoire of glycoenzymes with GOLPH3 (Welch et al., 2021). However, the role and function of GOLPH3L in glyco-oncology remains less well characterized.

### Adapters That Retain Enzymes in the Cisterna, or Retainers: GRASP55

GRASP55 was originally discovered as Golgi stacking protein (Shorter et al., 1999), but in recent years this role has been contradicted (Grond et al., 2020), and its function on the Golgi remains unclear. Our group showed that GRASP55 silencing causes a change in the localization of glycosphingolipids (GSL) synthesizing enzymes (Pothukuchi et al., 2021). GRASP55 interacts directly with glycoenzymes sharing consensus signals within their cytosolic tails, and excludes them from COPI recycling vesicles, resulting in the retention of its client enzymes in the maturing cisterna*,* like transported cargoes. This implies that a genetic deletion or mutation of GRASP55 in cancer might lead to an enhanced glycoenzyme recycling, increasing their levels by subtracting them from the normal lysosomal leakage and conferring a positional advantage in competition reactions, thereby affecting the glycosylation metabolic flux. Although there were no significant alterations at the genomic level at the GRASP55 locus in humans, mouse insertional mutagenesis studies have reported that GRASP55 inactivation promotes tumorigenesis in liver and colorectal cancer models (Ref: Candidate Cancer Gene Database). Thus, studying the role of GRASP55 in cancer through its role in the regulation of glycan synthesis might help to understand the role of aberrant glycan synthesis in tumorigenesis.

## Small GTPases as Molecular Switches of Cisternal Maturation

### Rab1

Rab-GTPases have long been known to localise to the Golgi complex (Martinez et al., 1994; Progida, 2019; Westrate et al., 2020). By cycling between their GTP-loaded active and GDP-loaded inactive states, Rabs spatiotemporally coordinate the recruitment of effectors to execute the various steps of Golgi cisternal progression and maturation. Indeed, recently, the Rab cascade has been proposed to drive maturation of the *cis-* into the *trans*-Golgi compartment in yeast (Kim et al., 2016).

There are close to 60 Rabs encoded in the human genome of which nearly two dozen are localized to the Golgi apparatus (Martinez and Goud, 1998; Goud et al., 2018). Two isoforms of Rab1- Rab1a and Rab1b are encoded in the human genome. Rab1 isoforms are mainly localised to the ER-Golgi intermediate compartments (ERGIC) and the *cis*-Golgi where they interact with golgins p115, GM130 and other Golgi proteins (Allan et al., 2000; Moyer et al., 2001). However, there are several studies in which Rab1b has been reported to interact with *trans*-Golgi localized proteins (Halberg et al., 2016), which suggest a localization and a role for Rab1b in the late Golgi cisterna as well. Rab1a was recently identified to be a colorectal oncogene whose expression is correlated with tumor invasion, poor prognosis and hyperactive mTORC1 signalling (Thomas et al., 2014). Further, Rab1a overexpression due to genetic amplification (Figure 5) has been strongly correlated to hepatocellular carcinoma progression (Xu et al., 2015). Rab1a is the first known Rab-GTPase to activate mTORC1 at the Golgi complex upon stimulation by amino acids and this function was conserved from yeast to mammals (Thomas et al., 2014; Xu et al., 2019). There is no published study linking Rab1 to altered glycosylation especially in cancers. However, Rab1 has been shown to interact with well-known regulators of glycosylation, such as GOLPH3. Indeed, in *Drosophila melanogaster* a direct *in vitro* interaction between Rab1 and GOLPH3 was shown, suggesting a Rab1-dependent regulation of GOLPH3 function (Sechi et al., 2017; Cavieres et al., 2020). However, in mammalian system the interaction between Rab1 and GOLPH3 is yet to be confirmed. In addition to promoting GOLPH3 recruitment by direct interaction, Rab1b at least can also promote this in an indirect manner. As mentioned earlier, GOLPH3 recruitment to the Golgi depends on PI(4)P levels and Rab1b plays an important role in the recruitment of PI4KIIIβ to Golgi membranes which then produces PI(4)P required for GOLPH3 recruitment. Indeed, overexpression of phosphatidylinositol (PI) transfer protein cytoplasmic 1 (PITPNC1) leads to Rab1b mediated recruitment of GOLPH3 to the Golgi (Halberg et al., 2016). Thus, by regulating the recruitment and likely the activity of the glycosylation regulator GOLPH3, Rab1 can regulate glycosylation function of the cell. Moreover, Rab1 also regulates recruitment of COPI coatomer by Arf1 on the Golgi membranes (Alvarez et al., 2003; Bannykh et al., 2005). Rab1b was shown to directly interact with GBF1 thereby stabilising Arf1-mediated COPI dynamics at the Golgi membrane (Monetta et al., 2007). Thus, Rab1 also regulates the formation of COPI vesicles, crucial for cisternal maturation, which in turn regulates glycosylation enzyme levels and localization within the Golgi. Thus, by regulating both enzyme sorting into and the formation of COPI vesicles, Rab1 has the potential to control glycosylation. Whether this is really the case and whether this can contribute to the oncogenic function of Rab1 remains to be experimentally tested.

### Arf1

Arf proteins belong to the Arf/Sar/SRβ family of small GTPases (Jackson and Casanova, 2000). There are six mammalian Arf family members of which five localize to the Golgi apparatus (Bonifacino and Glick, 2004; D'Souza-Schorey and Chavrier, 2006). Similar to Rab-GTPases, Arfs cycle between their GDP-bound inactive and GTP-bound active form on Golgi membranes to 1) enable membrane recruitment of different coat proteins to allow sorting of cargoes and the intra-Golgi transport of glycoenzymes (Jackson and Casanova, 2000), 2) indirectly define the lipid composition of the Golgi (De Matteis et al., 2007), 3) assemble a Golgi-associated cytoskeletal scaffold (Cao et al., 2005), and 4) modulate the post-Golgi trafficking to the PM (Cao et al., 2005). Of the five Arf proteins localized to the Golgi, Arf1 has been associated with oncogenesis. Amplification of Arf1 (Figure 5) has been found in 2.3% of cancer patients belonging to different subtypes and associated with poor prognosis of breast cancer patients (Xie et al., 2016). Arf1 overexpression promotes growth and metastasis in breast cancer, myeloma cells and ovarian tumors (Haines et al., 2015; Gu et al., 2017). Arf1 silencing and its pharmacological inhibition by LM11 compound, has been shown to suppress migration and invasion of breast and prostate cancer cell lines (Viaud et al., 2007). Arf1 also regulates migration by controlling the assembly of focal adhesions, presumably by regulating the glycosylation of their structural components (Schlienger et al., 2015; Casalou et al., 2016). The mechanism by which Arf1 alteration contributes to oncogenesis is not clear. We will discuss here two potential mechanisms by which Arf1 can contribute to oncogenesis through altered glycosylation.

Arf1 is recruited to different Golgi sub-domains by specific GEFs (Gustafson and Fromme, 2017) (Figure 4). At the *cis-*Golgi, Arf1 recruitment depends on the Arf-GEF, GBF1. Once recruited, Arf1 recruits COPI coatomer by binding directly to the β- and γ-COP subunits (Zhao et al., 1997; Zhao et al., 1999). Studies have demonstrated that growth factor mediated activation of the well-known oncogene Src kinase phosphorylates GBF1 which activates it to promote Arf1 mediated COPI recruitment and enhanced retrograde transport from Golgi to ER (Chia et al., 2021). This enhanced transport promotes the translocation of GALNT1 and GALNT2 to the ER via COPI recycling vesicles. It is likely that GALNT1 and GALNT2 directly bind to a specific subunit of COPI for their Arf1-dependent recycling toward the ER within COPI vesicles. Indeed, other GALNTs have been already demonstrated to interact with the δ- and ζ1-COPI through the ϕ(K/R)xLx(K/R) motif present in their cytosolic tails for their intra-Golgi recycling (Liu et al., 2018). The translocation of GALNT1 and GALNT2 to the ER increases the synthesis of Tn antigen [N-acetylgalactosamine (GalNAc) monosaccharide linked to Ser and Thr protein residues] (Gill et al., 2010), which is not only a well-known marker of cancers, but has also been shown to contribute to oncogenesis (Nguyen et al., 2017).

At the *trans-*Golgi, the Arf1-GEFs BIG1/2 enhance the membrane-association of Arf1-GTP within GORAB-Scyl1 oligomers. This facilitates the efficient recruitment and assembly of COPI coatomers to specific membrane sub-domains of the *trans*-Golgi (Zhao et al., 2002). Loss of GORAB or Scyl1 leads to COPI disassembly from the Golgi membranes leading to ST6Gal1 mislocalization resulting in terminal α2,6-sialylation defects (Witkos et al., 2019). Whether the converse, that is an increase in Arf1 levels, leads to enhanced sialylation usually associated with cancers requires experimental exploration.

## Phosphoinositides Metabolism: The PI4KIIIβ and PITPNC1 Prototypes

### PI4KIIIβ

The PI(4)P is the predominant phospholipid enriched at the Golgi complex. Together with Arf1, PI(4)P levels cooperate to regulate the process of intra-Golgi recycling of glycoenzymes. Indeed, it is shown that Arf1 and PI4KIIIβ exist in a complex to regulate PI(4)P levels at the Golgi (Godi et al., 1999) (Figure 4). PI4KIIIβ, along with PI4KII⍺ are the main PI 4-kinases (PI4Ks) responsible for PI(4)P synthesis (Waugh, 2019). The product of PI4Ks PI(4)P plays multiple functions at the Golgi such as: 1) inducing vesicle biogenesis from late Golgi (Rahajeng et al., 2019), 2) Golgi targeting of adaptor proteins like GOLPH3 (Dippold et al., 2009), 3) recruiting Arfaptins (Cruz-Garcia et al., 2013) and lipid transport proteins such as PITPNC1, oxysterol binding protein (OSBP), FAPP1, FAPP2, and CERT with the aid of Arf1-GTPase (Mesmin et al., 2013), 4) recruiting Rab11-GTPase that regulates trafficking from TGN (de Graaf et al., 2004; Morrow et al., 2014), and 5) recruitment of adaptor proteins involved in TGN-to-endosomes trafficking (Bonifacino and Glick, 2004) (Wang et al., 2007). Altogether PI4KIIIβ, PI4KII⍺, PITPs, the PI(4)P phosphatase Sac1, and OSBP finely tune the Golgi PI(4)P levels in a precise spatiotemporal manner to coordinate cargo secretion coupled with the glycoenzyme recycling. Several malignancies show dysregulation of PI(4)P synthesis and recruitment of its effectors to the Golgi complex (Tokuda et al., 2014; Tan X. et al., 2020). Amplification of PI(4)P-effectors, such as GOLPH3 or PITPNC1, has been observed in a number of tumors to promote aberrant glycosylation of proteins and lipids essential for cell adhesion, and tumor invasiveness (Halberg et al., 2016; Isaji et al., 2019). Similarly, aberrant Golgi PI(4)P metabolism has been reported to enhance the secretion of extracellular matrix remodelling proteins thereby promoting angiogenesis and enhanced motility (Tokuda et al., 2014).

PI4KIIIβ has been reported to be amplified (Figure 5) and overexpressed in 4% of cancers (Curtis et al., 2012). An oncogenic role for PI4KIIIβ has been demonstrated in breast and lung adenocarcinomas bearing the chromosome 1q amplification, which occurs at the frequency of 14%. PI4KIIIβ co-occurs with KRAS driver mutations, and accelerates lung adenocarcinoma progression (Tan X. et al., 2020). In this genetic context, it has been shown that the deletion of PI4KIIIβ or inhibition of its catalytic activity decreases cancer cell proliferation and induces apoptosis, demonstrating its value as a therapeutic target. Alternatively, in a subset of 1q-amplified lung adenocarcinoma which develops tolerance to PI4KIIIβ inhibitors and addiction to Golgi PI(4)P synthesis, blocking PI4KII⍺ has emerged as a potential therapy (Shi et al., 2021). PI4KIIIβ and Sac1 are key regulators of the localization and abundance of PI(4)P on Golgi which is important for proper steady-state distribution of glycoenzymes. Sac1 silencing resulted in decreased cell-cell adhesion and increased cell migration in breast cancer cells, while the silencing of PI4KIIIβ showed opposite effects in a PI(4)P dependent fashion (Ijuin et al., 2016). Increased PI(4)P level might exert its oncogenic function through the regulation of glycosylation *via* GOLPH3 recruitment, which is known to promote malignant phenotypes by relying on PI(4)P-binding and regulating the glycoenzyme recycling at specific sub-Golgi compartment. Other PI(4)P effectors, recruited at the Golgi with the aid of Arf1, are PIPTNC1, CERT, OSBP and FAPP2 (Waugh, 2019). Arfaptins bind to TGN-specific PI(4)P pools via their PH domain, and significantly change the lipid composition of the TGN through increased levels of GSLs, cholesterol and sphingomyelin, which from the TGN are then transported to PM (Simons and Sampaio, 2011). On PM these compounds have been shown to have a role in cancer signalling (Wu et al., 2020). Hence, these findings indicate the impact of Arf1 and PI4KIIIβ amplifications on aberrant PI(4)P metabolism leading to increased recruitment of PI(4)P effectors which subsequently affect the glycosylation machinery. PI4KIIIβ was also found co-amplified with GOLPH3L within the 1q amplicon in several lung adenocarcinoma patients (Tan X. et al., 2020). GOLPH3L was also shown to bind to a wide range of Golgi enzymes belonging to several glycosylation pathways (Welch et al., 2021), highlighting its possible role in the regulation of intra-Golgi recycling of enzymes and most likely in the synthesis of pro-tumorigenic glycans in tumors harbouring 1q amplification.

### PITPNC1

PI synthesis occurs in the ER, and PI transfer proteins such as PITPNC1 play an important role in delivering PI to the Golgi apparatus acting at the ER-Golgi interface (Ashlin et al., 2021). PITPNC1 is known to contribute to several oncogenic effects such as tumor angiogenesis, metastasis, and malignant secretion in breast cancer, omental metastasis of gastric cancer and in the development of radio-resistance in colorectal cancer (Tan et al., 2018; Tan Y. et al., 2020). A study by Halberg et al. (2016) has shown PITPNC1 to be frequently amplified (across 2% of cancers) and responsible for the secretion of matrix metalloprotease 9 (MMP9), platelet-derived growth factor (PDGF) and HTRA1 (a serine protease involved in epithelial-to-mesenchymal transition) in malignant tumors. PITPNC1 amplification and overexpression in cancers (Figure 5) increases the PI and phosphatidic acid pool at the Golgi complex, which serves as the substrate for Golgi localised PI4Ks for the production of PI(4)P (Garner et al., 2012). Additionally, PITPNC1 is known to stimulate membrane-associated PI4-kinase activity *in vitro* [87] (Contreras et al., 2012). It was observed that PITPNC1 amplification leads to enhanced pro-tumorigenic secretion by increasing the Golgi pool of Rab1b and its effector GOLPH3 which then binds to MYO18A to promote vesicle budding from the Golgi. Given its role in lipid transfer across Golgi membranes, PITPNC1 is mainly thought to affect cancer cell secretion and sustain tumor growth (Png et al., 2012; Tan X. et al., 2020). Although a role for PITPNC1 in direct regulation of glycosylation is not known, it might affect PI(4)P dependent intra-Golgi recycling and positioning of glycoenzymes, affecting the cancer cell glycome through PI(4)P-GOLPH3 axis. Further studies will shed light on the involvement of PITPNC1 in the regulation of Golgi enzymes dynamics and glycosylation process in physiology and in the tumor context.

## Conclusion and Perspectives

In this review, we have discussed how components of CMM that are frequently amplified in cancers (Figure 5) might represent oncogenes that act by deregulating glycan synthesis. The intra-Golgi glycoenzyme recycling mediated by CMM (e.g., Arf1, PI4KIIIβ, PITPNC1, GOLPH3, and Rab1) might affect the position and levels of glycoenzyme, as shown for some of them, thereby altering glycosylation flux and impacting the overall glycan outcome. Aberrant glycans exposed on cancer cells, in turn, exert their tumorigenic functions by different mechanisms [reviewed elsewhere (Mereiter et al., 2019). GOLPH3 and Arf1 have been previously shown to contribute to tumorigenesis through altered glycosylation suggesting a plausible oncogenic mechanism shared by other trafficking components when amplified in cancer (Chia et al., 2021; Rizzo et al., 2021).

Currently, there are a few inhibitors directed against the glycan synthetic machinery, but none are utilised as anticancer drugs. Few glycoenzyme inhibitors are currently being evaluated as drugs in clinical trials, such as fluorinated sugars analogs (van Wijk et al., 2015). The compound T3Inh-1 was shown to specifically block GALNT3 to reduce breast cancer cell growth *in vitro* (Song and Linstedt, 2017), and Swainsonine, a glycoenzyme specific inhibitor, showed anticancer effects on patients, with low toxicity (Galustian et al., 1994; Goss et al., 1994). The GSL inhibitor Miglustat is regularly used in clinics for the treatment of congenital disorders of glycosylation (CDG) and lysosomal storage diseases (Patterson et al., 2007). There are no trials in which Miglustat is used for anticancer treatments despite the established role of GSL in tumor progression. Given the scarcity of approved inhibitors of the underlying synthetic machinery, current anticancer interventions generally target directly the aberrant glycoforms exposed by cancerous cells, as is the case of the approved drug Dinutuximab which targets the GD2 ganglioside expressed on neuroblastoma cells, or for specific CAR-T recognizing an aberrant O-glycoform on MUC1 which is still in clinical trials (Yu et al., 2010; Posey Jr et al., 2016). Aberrant glycosylation plays an important role in Trastuzumab resistance as observed in ErbB2-positive unresected advanced gastric cancer patients. ST6Gal1-dependent sialylation within ErbB2 N-glycan site was shown to block the mAb-binding ectodomain, thereby antagonizing the pro-apoptotic effects of Trastuzumab (Duarte et al., 2021). A better understanding of cancer heterogeneity based on the oncogenic role of CMM or glycoenzymes, will improve our comprehension of the role of glycans in cancer biology. We expect that a detailed understanding of the mechanism(s) by which the CMM influences the synthesis of onco-glycans will reveal 1) novel markers to aid in the clinical stratification of patients, 2) new molecular targets enabling the precise manipulation of the synthetic glycan machinery, and 3) new drugs that will act synergistically to current treatment regime to develop personalized targeted anticancer therapies.

Treatment of certain cancers might benefit from glycoenzyme specific inhibitors when an individual glycoenzyme is aberrantly expressed. Instead, alterations of components of the CMM impact entire enzymatic modules, each consisting of different types of enzymes. This condition might be not easily modified by inhibitors targeting single glycoenzymes, as multiple co-occurring onco-glycoforms might be simultaneously active. Indeed, altered level of individual glycoenzyme may play a modest role in influencing the final glycosylation flux. Whereas, altered levels of CMM components are expected to induce an extensive remodelling of glycosylation reactions, as the CMM modulates simultaneously the dynamics of several sequentially acting enzymes which constitute a processing module. In this scenario, gross variations in an individual enzyme level are required to alter the flux through its subset of reaction network to affect the final glycosylation outcome. Conversely, a small fluctuation in the level and position of each glycoenzyme belonging to the same processing module, as observed upon CMM gene amplification, can lead to a significant effect on the metabolic flux of glycosylation, when the entire glycoenzymatic module is enhanced. Mechanistically, CMM components are limiting factors wherein copy number alterations of any one component might simultaneously enhance the membrane bound fraction of others, thus enhancing the entire COPI-dependent recycling process (we expect this to be true also in the case of amplification of components of COPI-independent recycling machinery). Interestingly, the specific intra-Golgi recycling machinery for all Golgi glycoenzymes are not known. For instance, MGAT5 is a putative glyco-oncogene whose overexpression enhances the synthesis of β1-6 branched N-glycans linked to adhesion molecules and RTKs, thus promoting tumor growth (Granovsky et al., 2000; Lau et al., 2007). Intriguingly, the amplification of CMM genes responsible for MGAT5 dynamics might increase the levels of this enzyme, phenocopying the effect on glycosylation observed upon MGAT5 overexpression, and promoting cancer. Alternative regulatory mechanisms based on loss of function mutations within the CMM might affect intra-Golgi glycoenzyme dynamics. Loss of function mutations within the recycling machinery of EXT1 and EXT2, known to act as tumor suppressors regulating proteoglycan synthesis, might lead to enzyme mislocalization outside Golgi, thus promoting sarcomas (McCormick et al., 2000). Similarly, the loss of function mutations or genetic deletions of retainers, such as GRASP55 might lead to excessive intra-Golgi recycling of specific glycoenzymes, thereby affecting glycosylation, as already shown for a few glycoenzymes belonging to GSL metabolism (Pothukuchi et al., 2021).

Further efforts are needed to elucidate the oncogenic potential of amplified CMM components and discover novel mechanisms impacting onco-glycan synthesis. These components, which were overlooked for years, might represent key molecules for understanding cancer heterogeneity and, by linking glycomics and cancer, might aid in the patient stratification and establishing personalized treatments.
